# Involvement of polyphosphate kinase in virulence and stress tolerance of uropathogenic *Proteus mirabilis*

**DOI:** 10.1007/s00430-015-0430-1

**Published:** 2015-08-02

**Authors:** Liang Peng, Qiao Jiang, Jia-Yun Pan, Cong Deng, Jing-Yi Yu, Xiao-Man Wu, Sheng-He Huang, Xiao-Yan Deng

**Affiliations:** Department of Clinical Laboratory, The Second Affiliated Hospital of Guangzhou Medical University, Guangzhou, 510260 China; Intensive Care Unit, Guangdong 999 Brain Hospital, Guangzhou, 510510 China; Department of Microbiology, School of Public Health and Tropical Medicine, Southern Medical University, Guangzhou, 510515 China; Saban Research Institute, Childrens Hospital Los Angeles, Los Angeles, CA 90027 USA

**Keywords:** *Proteus mirabilis*, Urinary tract infection, Polyphosphate kinase, Virulence

## Abstract

*Proteus mirabilis* (*P. mirabilis*), a gram-negative enteric bacterium, frequently causes urinary tract infections. Many virulence factors of uropathogenic *P. mirabilis* have been identified, including urease, flagella, hemolysin and fimbriae. However, the functions of polyphosphate kinase (PPK), which are related to the pathogenicity of many bacteria, remain entirely unknown in *P. mirabilis*. In this study, a *ppk* gene encoding the PPK insertional mutant in *P. mirabilis* strain HI4320 was constructed, and its biological functions were examined. The results of survival studies demonstrated that the *ppk* mutant was deficient in resistance to oxidative, hyperosmotic and heat stress. The swarming and biofilm formation abilities of *P. mirabilis* were also attenuated after the *ppk* interruption. In vitro and in vivo experiments suggested that *ppk* was required for *P. mirabilis* to invade the bladder. The negative phenotypes of the *ppk* mutant could be restored by *ppk* gene complementation. Furthermore, two-dimensional gel electrophoresis and liquid chromatography–mass spectrometry were used to analyze the proteomes of the wild-type strain and the *ppk* mutant. Compared with the wild-type strain, seven proteins including TonB-dependent receptor, universal stress protein G, major mannose-resistant/Proteus-like fimbrial protein (MR/P fimbriae), heat shock protein, flagellar capping protein, putative membrane protein and multidrug efflux protein were down-regulated, and four proteins including exported peptidase, repressor protein for FtsI, FKBP-type peptidyl-prolyl cis–trans isomerase and phosphotransferase were up-regulated in the *ppk *mutant. As a whole, these results indicate that PPK is an important regulator and plays a crucial role in stress tolerance and virulence in uropathogenic *P. mirabilis*.

## Introduction

*Proteus mirabilis* (*P. mirabilis*) is an important pathogen that causes urinary tract infections (UTIs), especially in patients with indwelling urinary catheters [1]. A recent investigation of clinic UTIs caused by gram-negative bacilli found that *P. mirabilis*-induced UTI was third in infection rates after *Escherichia coli* and *Klebsiella pneumoniae* [2]. *P. mirabilis* colonization and invasion of the uroepithelium are two crucial steps in the pathogenesis processes of UTI. Some virulence factors related to these processes have been identified. Mannose-resistant *Proteus*-*like* (MR/P) fimbria, as an adhesin, is important for bacterial adhesion to the uroepithelium. The secreted hemolytic toxin HpmA contributes to host cell invasion and cytotoxicity [3]. Prior to the successful colonization of the urinary tract, pathogens need to overcome several challenges, including urine flow, antibacterial molecules, pH and osmotic pressure [4]. Recent research has suggested that the bacterial RNA chaperone Hfq plays a critical role in *P. mirabilis* adaptation to environmental stress and urinary tract colonization [5]. Flagella-mediated motility and RsbA-mediated fatty acid regulation are both involved in the swarming differentiation and migration, which are characteristics of *P. mirabilis* and related to UTIs caused by *P. mirabilis*.

Polyphosphate kinase (PPK), encoded by the *ppk* gene, is a major enzyme involved in the synthesis of inorganic polyphosphate (poly P) from ATP. Some studies have indicated that PPK plays an important role in the stress resistance and virulence of several bacteria species. The meningitis *E. coli* K1 strain with a *ppk* knockout was deficient in responses to stresses and translocation across the blood–brain barrier [6]. *Vibrio cholera ppk* mutants were defective in motility and cell surface attachment [7]. *Pseudomonas aeruginosa* exhibited aberrant quorum sensing and biofilm formation after *ppk* deletion [8]. Inactivation of *ppk* in *Shigella* and *Salmonellaspp.* led to loss of the capacities to resist environmental stress and to invade epithelial cells [9].

However, the function of *ppk* in *P. mirabilis* remains entirely unknown. In this study, we constructed a *ppk* mutant strain and investigated the role of *ppk* in UTIs caused by *P. mirabilis* using both in vitro and in vivo models. In addition, two-dimensional gel electrophoresis and liquid chromatography–mass spectrometry (LC–MS) were employed to analyze the molecular regulation mechanism of *ppk* in *P. mirabilis*.

## Materials and methods

### Bacterial strains, plasmids and growth conditions

*Proteus mirabilis* HI4320 (clinically isolated strain from a patient with UTI, Tet^r^) was kindly provided by Prof. Harry L. T. Mobley. *E. coli* TOP10 cells were used as the host for plasmid construction. All strains, including the wild-type strain, the mutant, and the ppk-complemented strain, were cultured at 37 °C in Luria–Bertani (LB) broth (10 g/L of tryptone, 5 g/L of yeast extract and 10 g/L of NaCl) and non-swarming agar (10 g/L of tryptone, 5 g/L of yeast extract, 5 mL/L of glycerol, 0.4 g/L of NaCl and 20 g/L of agar). Antibiotic supplementation with ampicillin (100 µg/mL), chloramphenicol (20 µg/mL) or kanamycin (25 µg/mL) was provided as necessary. The plasmids pACD4 K and pAR1219, used for *ppk* gene mutant construction, were purchased from Sigma-Aldrich. The rabbit polyclonal antibody against PPK protein was constructed in our laboratory.

### Construction of the isogenic insertional mutant

The mutant was constructed using the TargeTron system (Sigma-Aldrich, USA), according to the manufacturer’s instructions and as described as by Harry L. T. Mobley, et al. [10]. Briefly, a reprogrammed group II intron (containing a kanamycin resistance gene) was specifically inserted into the site of 201/202a in *ppk* via mutagenic PCR using the primers ppk-IBS, ppk-EBS2, ppk-EBS1d, and EBS universal (Table 1). The retargeted intron was ligated into plasmid pACD4 K-C (containing a chloramphenicol resistance gene) to create plasmid pACD4 k-ppk and was transformed into *E. coli* top 10 cells by electroporation. Then, the correct recombinant plasmid was introduced into *P. mirabilis* HI4320, which had been previously transformed with a T7 helper plasmid pAR1219 (containing an ampicillin resistance gene). IPTG (1 mmol/L) was used to induce the intron from pACD4 k-ppk to jump into the *ppk* gene in the *P. mirabilis* chromosome (chloramphenicol/ampicillin double-resistant). Successful intron insertion was detected by kanamycin selection. The insertional mutant was confirmed with PCR using the primers ppk-outF and ppk-outR (Table 1). The correct insertion position was sequenced using the primers EBS universal and Intron3end (Table 1). Finally, the successfully constructed *ppk* mutant was named as PPK4320.

**Table 1:** Primers used for the construction of the *ppk* mutant and *ppk* complementation strain

Primer name	Sequence (5′–3′)
ppk-IBS	AAAAAAGCTTATAATTATCCTTAAGAGCCTGAGCCGTGCGCCCAGATAGGGTG
ppk-EBS1d	CAGATTGTACAAATGTGGTGATAACAGATAAGTCTGAGCCGCTAACTTACCTTTCTTTGT
ppk-EBS2	TGAACGCAAGTTTCTAATTTCGATTGCTCTTCGATAGAGGAAAGTGTCT
ppk-outF	CAAATATCCCGGTCTGCATACACAT
ppk-outR	CAATACACCTTCACGTAATGCACCA
EBS universal	CGAAATTAGAAACTTGCGTTCAGTAAAC
Intron3end	CAGAGCCGTATACTCCGAGA
pGMF	ATAAGTACTTTGAGACAATTTACCGAACAAC
pGMR	ATAAGTACTAGAAATGCCTCGACTTC
pkF	ATAGATATCATGTCCCAAGAACGACTCTATATTGATAAAG
pkR	AGAGGATCCTTACGCTCGTGATCCTGGTTGTTC

### Construction of the ppk-complemented strain

Firstly, a gentamycin-resistant gene was amplified by using PCR from suicide vector pJQ200SK with the primers of pGMF and pGMR (Table 1) to obtain a selection gene. The amplified DNA fragment was inserted into the ScaI restriction enzyme sites of the ampicillin-resistant gene in pBR322 to generate pBR322Gm. Secondly, the *ppk* gene was amplified with the primers, pkF containing an EcoR V enzyme site and pkR containing a BamH I enzyme site (Table 1). Then, the DNA fragment was subcloned into the tetracycline resistance gene between the EcoR V and BamH I sites of pBR322Gm. At last, the recombinant plasmid was named as pBR322Gm-ppk and then transformed into the ppk knockout mutant to generate the ppk-complemented strain PPK4320C. The growth curves of the mutant strain PPK4320, wild-type strain and PPK4320C in Luria–Bertani (LB) broth medium were determined by measuring the optical density (OD) 600 nm at different points in time.

### Western blotting analysis of the PPK expression

The production of the PPK protein after *ppk* gene mutation and complementation was detected by Western blotting as follows: 10 ml of overnight cell culture was resuspended in 1 ml lysis buffer [lysis buffer (pH 8.0): 50 mM Tris–HCl, 2 mM EDTA, 100 mM NaCl, 0.5 % Triton X-100, lysozyme 0.1 mg/Ml, 1 mM PMSF]. The samples were sonicated in a ice bucket for 3 × 10 s and then centrifuged for 5 min at 13,000 rpm at 4 °C. Ten microliters of supernatant was used for protein quantification. The protein samples were separated by sodium dodecyl sulfate-polyacrylamide gel electrophoresis (SDS-PAGE) on a 10 % polyacrylamide resolving gel. Then, the electrophoresed proteins were transferred to a PVDF membrane and incubated for 1 h at room temperature with 5 % fat-free milk. The membrane was incubated overnight at 4 °C with primary antibody. After being washed at room temperature for 30–60 min with 5 or more changes of PBST wash buffer, the membrane was incubated for 1 h at room temperature with goat anti-rabbit IgG-HRP secondary antibody. Then, the membrane was washed for 30–60 min with 5 or more changes of PBST wash buffer. Finally, the member was detected with chemiluminescent detection substrate and exposed to film. Then, its image was developed. 

### Measurement of the poly P levels

Detection of poly P levels in the wild-type, PPK4320 and PPK4320C was performed as described previously [11]. Bacteria were grown in LB medium at 37 °C for 18 h. Triplicate 1 ml aliquots of cells were collected and pelleted by centrifugation at 10,000 rpm for 2 min. To the pellets was added 500μL of 4 M guanidine isothiocyanate (GITC) lysis buffer (4 M GITC, 500 mM Tris–HCl, pH 7.0) prewarmed at 95 °C. The pellets were vortexed, incubated for 5 min at 95 °C and sonicated briefly. Protein quantitation was performed by using the Protein Quantitation Kit (TIAN GEN Biotechnology Co. Ltd, China). Then, to each tube, 30 μL of 10 % sodium dodecyl sulfate, 500 μL of 95 % ethanol and 10 μL of glassmilk (Bio 101, CA, USA) were added. After being vortexed, the tube was centrifuged briefly, and then, the pellet was suspended in 500 μL of cold New Wash buffer (5 mM Tris–HCl [pH 7.5], 50 mM NaCl, 5 mM EDTA and 50 % ethanol). This step was repeated three times. The washed pellet was resuspended in 50 μL of 50 mM Tris–HCl pH 7.4, 10 mM MgCl_2_ and 20 μg each of DNase and RNase per milliliter and incubated at 37 °C for 10 min. The pellet was first washed with 150 μL of 4 M GITC lysis buffer and 150 μL of 95 % ethanol and then twice in New Wash buffer. Poly P was eluted from the glassmilk pellet with 50 μL of 50 mM Tris–HCl (pH 8.0) at 95 °C for 2 min, followed by two additional elutions. Subsequently, a toluidine blue O (TBO) method was used to measure the poly P levels. A standard poly P curve was made by serially diluting a solution of a known quantity of sodium poly P (Sigma-Aldrich) and adding 100 μL aliquots to 900 μL of 6-mg/L TBO dye (Sigma-Aldrich) in 40 mM acetic acid. After incubation at room temperature for 15 min, OD530 nm and OD630 nm were measured, and the amount of poly P was expressed as the ratio of OD530 nm to OD630 nm. The poly P concentrations of wild-type, PPK4320 and PPK4320C were measured by comparing the ratio of OD530 nm to OD630 nm with the standard curve. Poly P quantity in cells was expressed in micrograms of poly P per milligram of total cellular protein.

### Stress tolerance assays

Bacteria were grown for 18 h in LB at 37 °C. For the oxidative stress assay, cells were collected and washed with phosphate-buffered saline (PBS) and then diluted to an OD600 of 1.0. H_2_O_2_ was added into the bacterial suspension at final concentrations of 0, 20, 40, 60, 80 and 100 mmol/L and incubated at 37 °C for 15 min. Then, 100 µL of samples was withdrawn, diluted in PBS and plated on non-swarming LB agar plates to determine viable cell numbers. For the osmotic challenge, bacteria were collected, suspended and diluted to 10^7^ cells/mL in PBS. Then, the samples were mixed with an equal volume of 4.8 mol/L NaCl (final concentration: 2.4 mol/L) and incubated at 37 °C. Samples collected at 0,  0.5, 1, 1.5 and 2 h were diluted and plated on non-swarming LB agar plates to determine viable cell numbers. For the heat resistance test, the PPK4320, PPK4320C and wild-type bacteria were centrifuged, resuspended in PBS and incubated at 56 °C for 0, 2, 4, 6 and 8 min. Samples were collected at the different time points, diluted and plated on non-swarming LB agar plates to determine viable cell numbers. All survival rates under stress conditions were determined by the number of viable cells after exposure to stress divided by the viable cell numbers before exposure to stress [5, 6].

### Cell adhesion and invasion assays

The 5637 cells (human urothelial bladder cell line) were grown in 1640 medium supplemented with 10 % heat-inactivated fetal bovine serum, penicillin G (50 μg/mL) and streptomycin (100 μg/mL) at 37 °C in 5 % CO_2_. The adhesion assay was performed according to previously reported methods [12]. Confluent monolayers of 5637 cells in 24-well plates (approximately 10^5^ cells/well) were incubated with bacteria [10^7^ colony-forming units (CFU)/well] for 3 h. After incubation, the monolayers were washed four times with PBS and then suspended in 0.5 % Triton X-100 for 8 min. The numbers of bacteria were counted on non-swarming agar plates. Adhesion was expressed as the percentage of adherent bacteria versus total bacteria added.

For the invasion assay, the invasion of 5637 cells by bacteria was performed as described in a previous study [6, 13]. Briefly, confluent monolayers of epithelial cells in 24-well plates (10^5^ cells/well) were incubated with bacteria (10^7^ CFU/well) for 1.5 h. The monolayers were washed with PBS and then incubated with non-serum 1640 medium containing gentamicin (100 μg/mL) for another 1, 2 and 3 h at 37 °C to eliminate the extracellular bacteria. To determine the number of intracellular bacteria, the monolayers were washed with PBS and lysed with 0.5 % Triton X-100. The released intracellular bacteria were determined by serial dilution and plating on non-swarming agar plates. The results are expressed as the percentage of viable bacteria that survived the gentamicin treatment versus the total bacteria added.

### Mouse model of UTI

The C57BL/6 mouse model of UTI was established according to methods described previously with minor modifications [5, 14]. Briefly, 7-week-old female C57BL/6 mice were deeply anesthetized with an intraperitoneal injection of 10 % chloral hydrate (4 µL/g). Prior to infection, the urinary bladders of the mice were voided by gentle compression of the abdomen. Then, the mice were inoculated transurethrally with a 50 µL suspension of 2–5 × 10^10^ CFU/mL of the wild-type strain, PPK4320, or PPK4320C using a venous indwelling needle (24G) and a microinjector (100 µL). Controls were injected with sterile PBS. At 48 h post-infection, the bladders of the mice were aseptically removed, divided into two parts and weighed. One part of the bladder was homogenized in 0.5 mL sterile PBS and was used for viable colony-forming unit counts and an inflammatory factors test using enzyme-linked immunosorbent assay (ELISA). The other portion was used for histological analysis. The viable counts were determined as CFU per gram of bladder tissue. The limit of detection of viable CFU counting was 10^2^ CFU/g bladder tissue. This animal study was approved and performed in accordance with guidelines of the Committee for Animal Studies at Guangzhou Medical University.

### Cytokine testing and histological analysis

One part of the collected bladder tissue was homogenized and 100 µL of the homogenate was used for viable counts determination. The remaining homogenate was centrifuged at 10,000 rpm for 10 min. The supernatant was collected and used for an ELISA determination of the inflammatory factors tumor necrosis factor-alpha (TNF-α) and interleukin-6 (IL-6), according to the manufacturer’s instructions (MultiSciences (Lianke) Biotech). The other part of the bladder was immersion-fixed overnight in 4 % paraformaldehyde, embedded in paraffin, cut into 5-μm-thick sections and stained with hematoxylin and eosin for histological evaluation.

### Swarming and biofilm formation assays

The swarming assay was performed as described in a previous study [15]. Briefly, 5 µL of a bacteria culture was inoculated in the center of the surface of swarming plate agar (containing 1.5 % W/V agar and 5 % V/V defibrinated sheep blood) and incubated at 37 °C. The swarming migration distances were quantified by following the swarm fronts of the wild-type strain and *ppk* mutant cells and recording the progress at 1-h intervals.

The biofilm assay was performed as described by Esther Heikens et al. with minor modifications [16]. In brief, bacteria were cultured in LB medium for 18 h at 37 °C with shaking. Then, for each well of a 96-well polystyrene microtiter plate, two hundred µL of LB medium mixed with 10 µL of (1 × 10^7^ CFU) cultured bacterial suspension were added in triplicate and incubated for 12 h at 37 °C. Every 2 h after incubation, the bacteria were removed and the wells were washed with 200 µL PBS. The plates were dried for 1 h at room temperature. The bacteria were fixed with methanol for 30 s. Then, 100 µL of 1 % Gram’s crystal violet solution was added to each well. After 30 min, the stain was removed and the plates were washed three times with 200 µL of PBS. The Gram’s crystal violet was extracted with a 100 µL solution of ethanol/acetone mixture (volume of ethanol: acetone = 80:20). Two hundred µL of extraction samples was added into the new wells of the 96-well plate. The absorbance at 570 nm was measured directly with an ELISA reader.

### Proteome analysis

Sample Preparation. Protein extraction and two-dimensional gel electrophoresis (2-DE) were performed using methods described previously [12, 17] with minor modifications. Briefly, *P. mirabilis* H4320 and the mutant PPK4320 were cultured in LB medium for 18 h with shaking. The bacterial cells were harvested by centrifugation at 8000rcf for 10 min. The pellets were mixed with lysis buffer containing protease inhibitor and lysozyme. Then, the mixtures were lysed using an ultrasonic wave (5 s on/5 s off at 37 % intensity for 5 min) and held in an ice bath. Samples were centrifuged at 20,000 rcf for 45 min at 4 °C, and the supernatants were collected. The protein samples were purified with an Amershan cleanup kit and quantitated with a GE 2-D Quant Kit (according to the manufacturer’s instructions).

2-DE. The protein extraction was dissolved in rehydration buffer (7 M urea, 2 M thiourea, 4 % CHAPS3 [w/v], 40 mM DTT, 1 % pH 4–7 immobilized pH gradient [IPG] buffer and 1 % bromophenol blue solution). The first and second dimensions of the PAGE were performed at least three times. Solubilized total protein samples (450μL each) were loaded onto 24-cm IPG strips (pH 4–7). The rehydration and isoelectric focusing (IEF) were performed according to the manufacturer’s instructions using an Ettan IPGphor II apparatus (Amersham Biosciences, Sweden). The second-dimension SDS-PAGE was performed with 12.5 % resolving gels and 5 % stacking gels using an Ettan DALT six instrument (Amersham Biosciences). The gels were stained with staining solution (Coomassie R-350 0.025 % and Glacial acetic acid 10 %). The resolved proteins in gels were scanned using an ImageScanner II (Amersham Biosciences) and were analyzed with ImageMaster V5.0 software. Only those protein spots with differences in density of 1.5-fold or greater between the groups were selected. In-Gel Protein Digestion. Protein bands were excised from preparative Coomassie blue-stained gels and washed several times with destaining solutions (25 mM NH_4_HCO_3_ for 15 min and then 50 % acetonitrile containing 25 mM NH_4_HCO_3_ for 20 min at 37 °C). Then, gel pieces were dehydrated with 100 % acetonitrile. Once dried, 10 μL of 12.5 ng/µL trypsin diluted in 25 mM NH_4_HCO_3_ was added over the gel spots, and the solution was incubated for 45 min at 4 °C. The redundant trypsin solution was absorbed away, and 10μL of 25 mM NH_4_HCO_3_ was added over the gel spots and incubated overnight at 37 °C.

#### Protein identification

Extraction was performed in two steps by the additions of 50 % acetonitrile and 0.1 % trifluoroacetic acid. The pooled extract was dried and dissolved in a 3 μL matrix (5 mg/mL recrystallized α-cyano-4-hydroxy-cinnamic acid) and spotted on an MS sample plate. Mass analysis was performed using an Ultraflex III-MALDI TOF/TOF Mass Spectrometer (Bruker, Germany). The peptide mass spectrum was obtained in positive ion reflector mode. Monoisotopic peak masses were limited within the range of 700–3500 Da. Trypsin autolysis peptides of mass 842.5 were used as internal standards. Four of the most intense ion signals were automatically selected as precursors for MS/MS acquisition, excluding the trypsin autolysis peaks and matrix ion signals. The peptide mass fingerprint combined with the MS/MS spectra data was searched against the NCBI database using Biotools software and MASCOT (Matrix Science).

### Statistical analysis

All values are expressed as mean ± standard deviation. Statistical analysis was performed with one-way ANOVA by using SPSS 13.0 software. Differences with *P* < 0.05 were considered to be statistically significant.

## Results

### *ppk* is important for poly P synthesis of *P. mirabilis*

**Fig. 1:**
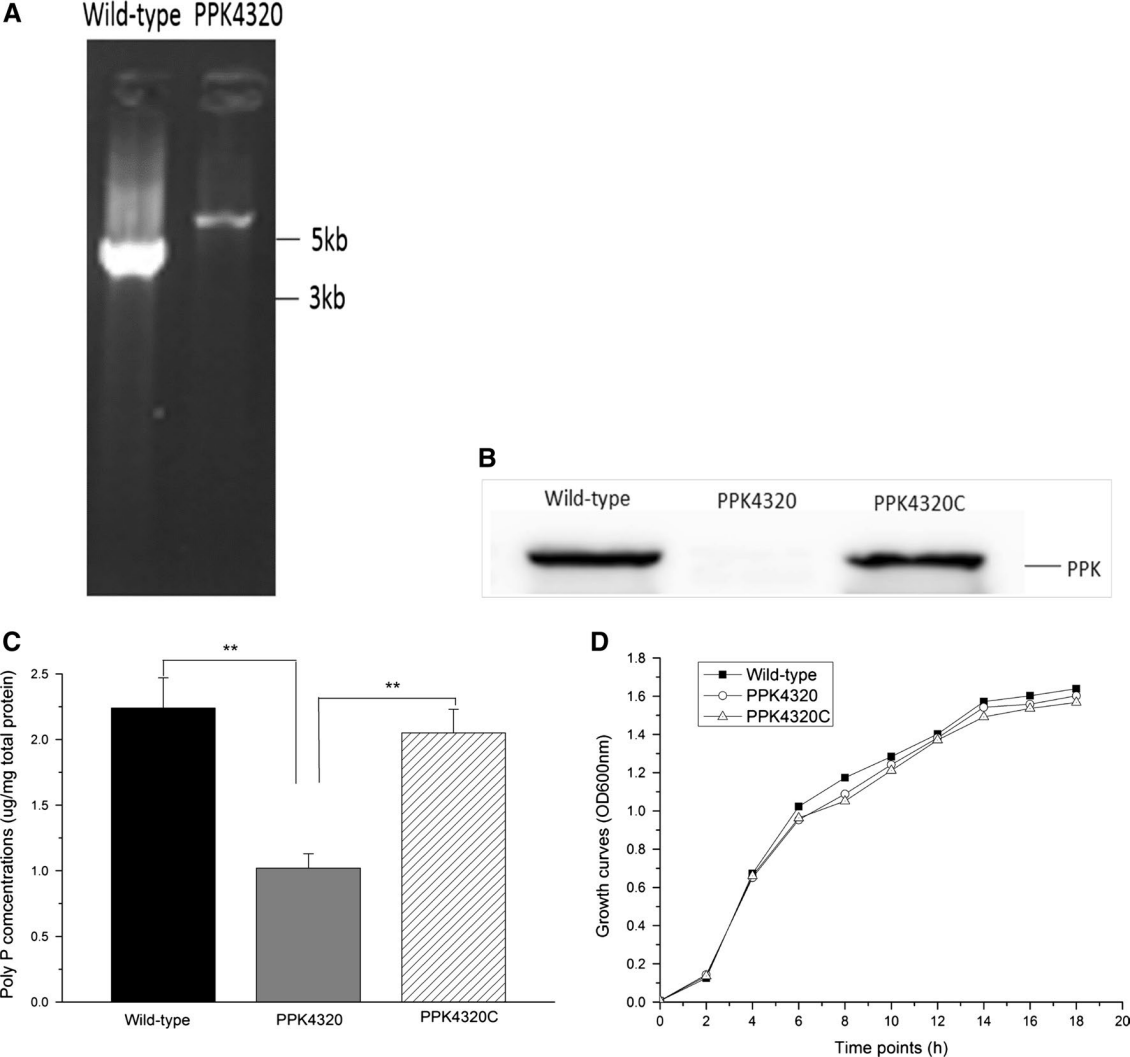
PPK protein and poly P production of the wild-type, PPK4320 and PPK4320C. Bacteria total protein was extracted from overnight (18 h) LB broth cultures. **a** PCR verification of intron insertion with the primers ppk-outF and ppk-outR. **b** Western blot testing of the PPK protein expression. **c** Poly P levels analysis. Values are expressed as the quantity of poly P (μg) per milligram of total cellular protein. **d** Examination of the growth curves. *P < 0.05, **P < 0.01

To determine the role of PPK in the pathogenesis of *P. mirabilis* UTI, the *ppk* gene was inactivated by insertion of a kanamycin cassette (about 2 kb). As shown in Fig. 1a, the size of PCR-amplified DNA fragment for the wild-type and mutant strain was about 4 and 6 kb, respectively. This result suggested that the intron had been inserted into the *ppk* gene successfully. DNA sequencing result suggested that the intron was inserted at a correct location (data not shown). The PPK protein production was shown to be an undetectable level in the *ppk* mutant by Western blot analysis. After transformation with the *ppk* complementation plasmid, the PPK expression of the *ppk* mutant was almost recovered to a level of the wild-type (Fig. 1b). PPK has been shown to be associated with the poly P synthesis [6, 9]. Similar results were obtained through detection of poly P. As shown in Fig. 1c, when the *ppk* gene was disrupted, the poly P level of the *P. mirabilis* declined significantly. The mutant PPK4320 and the *ppk* complementation strain PPK4320C showed no significant growth defects in vitro compared to the wild-type (HI4320) when the three strains were grown independently in LB medium (Fig. 1d).

### *ppk* is important for stress tolerance of *P. mirabilis*

**Fig. 2:**
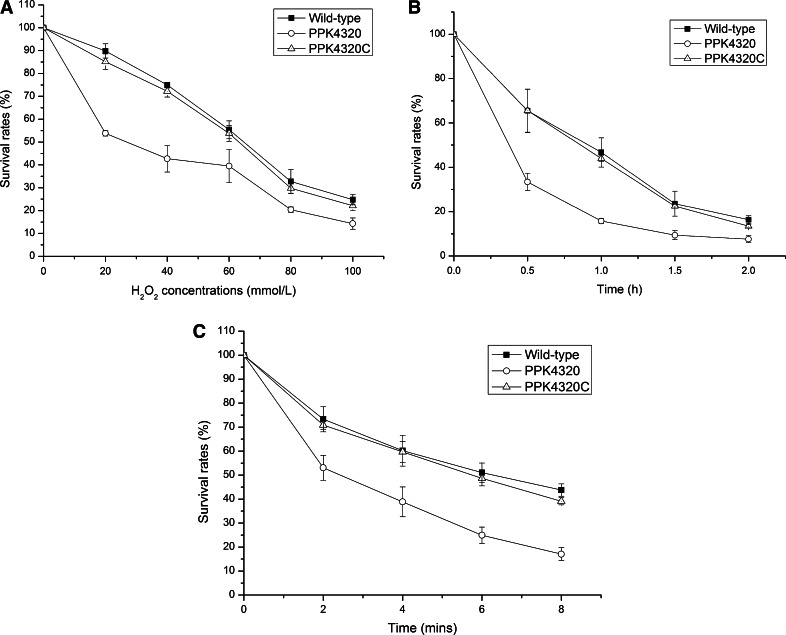
Survival assays. **a** The survival rates of the *ppk* mutant PPK4320 compared with the parent strain *P. mirabilis* HI4320 and *ppk* complementation strain PPK4320C after exposure to oxidative stress. **b** The survival rates of the PPK4320 compared with those of the wild-type and PPK4320C under a high osmolarity challenge. **c** The survival rates of the PPK4320 compared with the wild-type bacteria and PPK4320C under heating conditions

After exposure to H_2_O_2_ at different concentrations for 15 min, the survival rates of PPK4320 were significantly lower than those of the wild-type and PPK4320C (*P* < 0.01) (Fig. 2a). To test the survival under osmotic stress, bacteria were exposed to 2.5 mol/L NaCl for 0.5, 1, 1.5 and 2 h and then plated on LB agar plates. The survival rates of HI4320 were 65.78 ± 9.77, 46.72 ± 6.60, 23.59 ± 5.64 and 16.37 ± 1.82 % at 0.5, 1, 1.5 and 2 h, respectively. However, the survival rates of PPK4320 reduced to 33.38 ± 3.80, 15.76 ± 1.20, 9.41 ± 2.00 and 7.61 ± 1.66 % at 0.5, 1, 1.5 and 2 h, respectively (*P* < 0.01 when compared with HI4320 and PPK4320C). The survival rates of PPK4320C were restored to 65.54 ± 9.68, 43.97 ± 1.26, 22.46 ± 0.85 and 13.45 ± 0.73 % at 0.5, 1, 1.5 and 2 h, respectively (Fig. 2b). For the heat resistance test, PPK4320 and the wild-type strain were incubated at 56 °C for 0, 2, 4, 6 and 8 min. Similar to the results of oxidative and osmotic stress tests, the *ppk* mutant was deficient in survival under heat stress conditions compared with the wild-type and PPK4320C (*P* < 0.01) (Fig. 2c). These results suggest that *ppk* is important for *P. mirabilis* HI4320 to resist environmental stresses.

### *ppk* mutant is deficient in adhesion to and invasion of uroepithelial cells

**Fig. 3:**
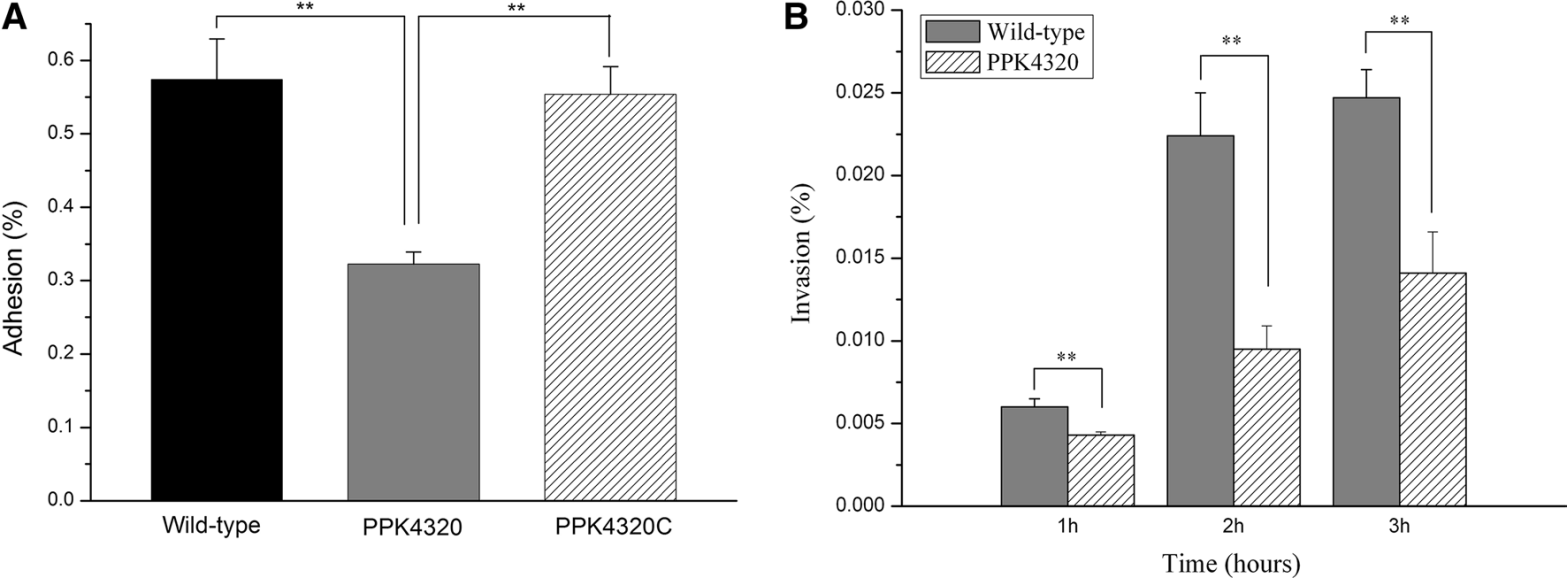
Wild-type, PPK4320, or PPK4320C adhesion or invasion of 5637 cells. **a** 5637 cell monolayers were incubated with equivalent amounts of the parent strain, the PPK4320 and PPK4320C for 3 h. A standard adhesion assay was performed as described in the “Materials and methods” section. **b** Invasion of 5637 cells for the wild-type strain or PPK4320 at 1, 2 and 3 h. Values are the means of at least three independent assays. *Error bars* indicate standard deviations. **P < 0.01

Adhesion to uroepithelial cells and subsequent colonization of tissues are important for *P. mirabilis* to resist urine flow and to establish a further infection. Invasion of the cells is helpful for *P. mirabilis* to evade immune attacks and to persist in tissues. So the effects of the *ppk* mutation on *P. mirabilis* adhesion and invasion of bladder uroepithelial cells were examined. As shown in Fig. 3a, the adhesion of uroepithelial cells for PPK4320 was significantly less than that of the wild-type or PPK4320C (*P* < 0.01). Furthermore, the capability of PPK4320 to invade uroepithelial cells was also lower than that of the wild-type strain at 1, 2 and 3 h, respectively (*P* < 0.01) (Fig. 3b).

### *ppk* inactivation leads to a less colonization of bladder tissue in mouse models for *P. mirabilis* HI4320

**Fig. 4:**
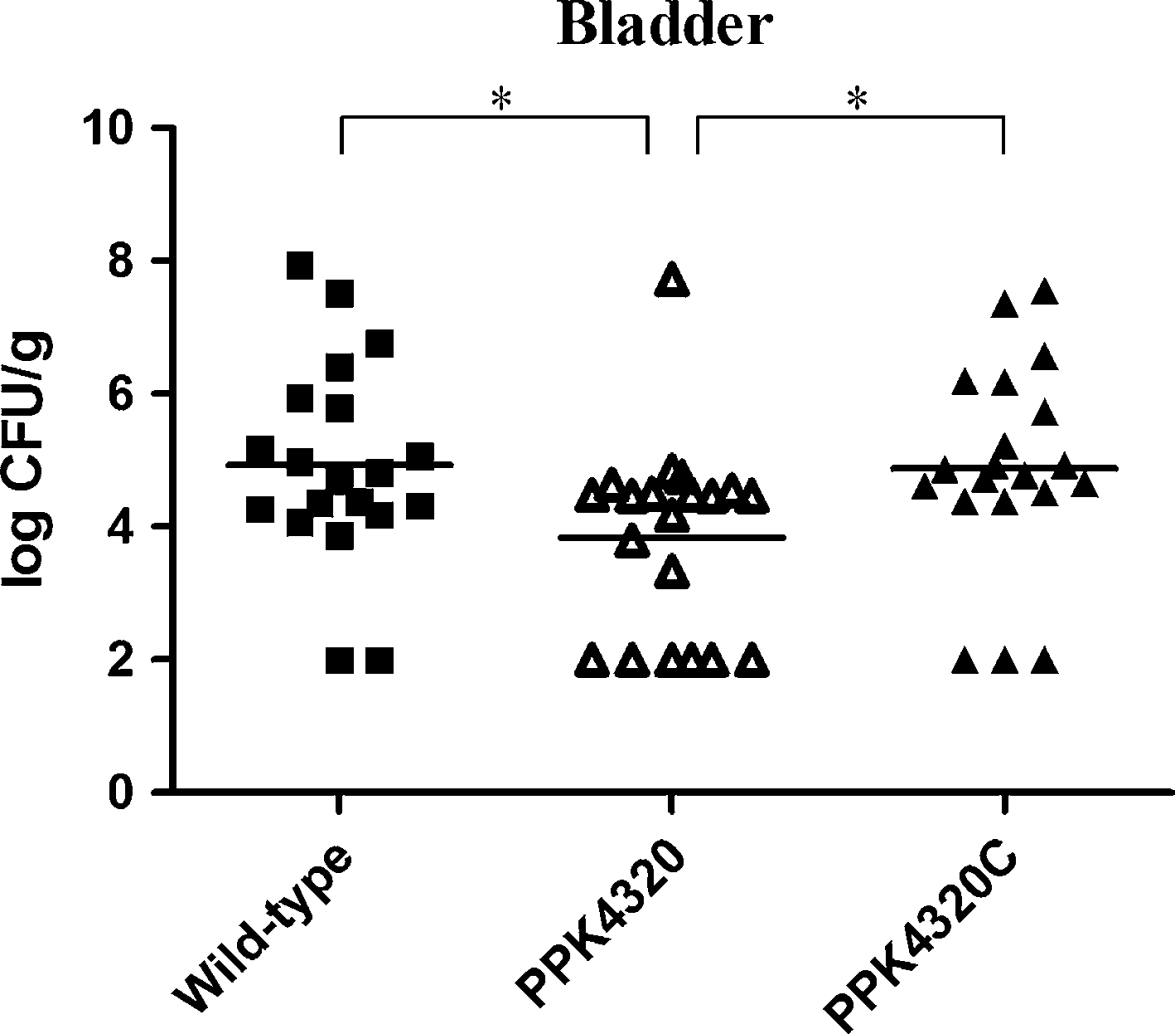
Quantitative bacterial counts in bladders of mice challenged with either the wild-type, PPK4320 and PPK4320C. Each *square* or *triangle* represents CFU per gram of bladder tissue from an individual mouse. *Horizontal lines* represent the geometric means of the colony counts. *P < 0.05

To evaluate the role of *ppk* in UTIs caused by *P. mirabilis*, female C57BL/6 mice were used as the in vivo study model. As shown in Fig. 4, after 48 h of inoculation with PPK4320 for 20 mice, only 14 mice had the level of bacterial colonization more than 10^2^ CFU/g bladder tissue. However, seventeen or eighteen mice had this level of colonization in twenty mice infected with PPK4320 or the wild-type strain, respectively. The amounts of bacteria colonization in bladders of the PPK4320-infected mice were also significantly less than those of the wild-type-infected or PPK4320C-infected animals. These results indicate that *ppk* is important for the colonization and survival of *P. mirabilis* in the bladder.

### The *ppk* gene is important for *P. mirabilis* to induce urinary tract inflammation in mice

**Fig. 5:**
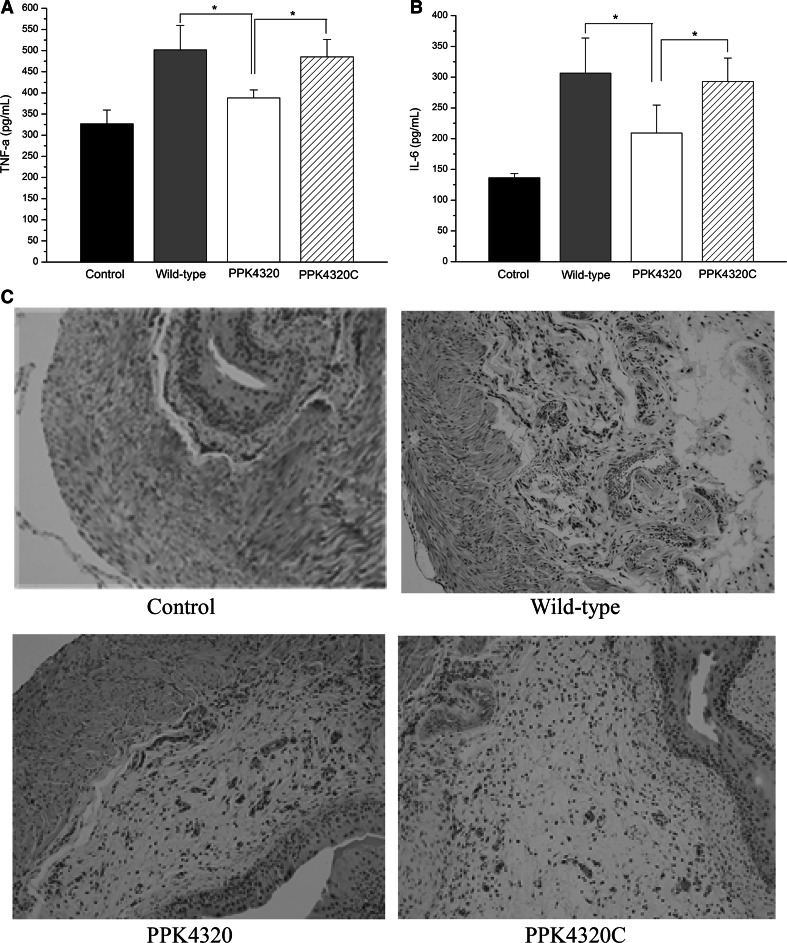
Inflammatory cytokine determinations and histological analysis of the bladder tissues in C57BL/6 mice infected with *P. mirabilis*. **a** TNF-α levels in the bladders of the mice. **b** IL-6 levels in the bladders of the mice. **c** Histological analysis of the bladder sections. The cytokine examination and histological analysis were performed as described in the “Materials and methods.” *P < 0.05

Because TNF-α and IL-6 are both important cytokines contributing to the host immune system against bacterial infection [18–20], we examined these cytokines in the bladder tissues of the experimental mice using the ELISA method. As shown in Fig. 5a, the TNF-α level in bladders of the wild-type-infected mice (501.70 ± 57.86 pg/mL) and PPK4320C (485.22 ± 40.91 pg/mL) was significantly higher than those of mice infected with the *ppk* mutant (388.13 ± 19.03 pg/mL). Additionally, the IL-6 levels in the mutant-challenged group (209.00 ± 45.72 pg/mL) were reduced compared to the wild-type-infected animals (306.63 ± 56.98 pg/mL) and PPK4320C (292.81 ± 38.3 pg/mL) (Fig. 5b).

Moreover, histologic examination of the bladders with hematoxylin and eosin staining also indicated that *ppk* was correlated with inflammation in *P. mirabilis*-induced UTI. As shown in Fig. 5c, the bladder submucosa of mice with the wild-type or PPK4320C infection became obviously hydropic and hyperemic, and the mucous layers had large numbers of neutrophil infiltrations. In contrast, less histological alteration was observed in the bladder tissues of the mice infected with the *ppk* mutant. These results indicate that *ppk* plays a key role in the inflammation response of *P. mirabilis*-induced UTI.

### *ppk* mutation affects swarming and biofilm formation abilities of *P. mirabilis*

**Fig. 6:**
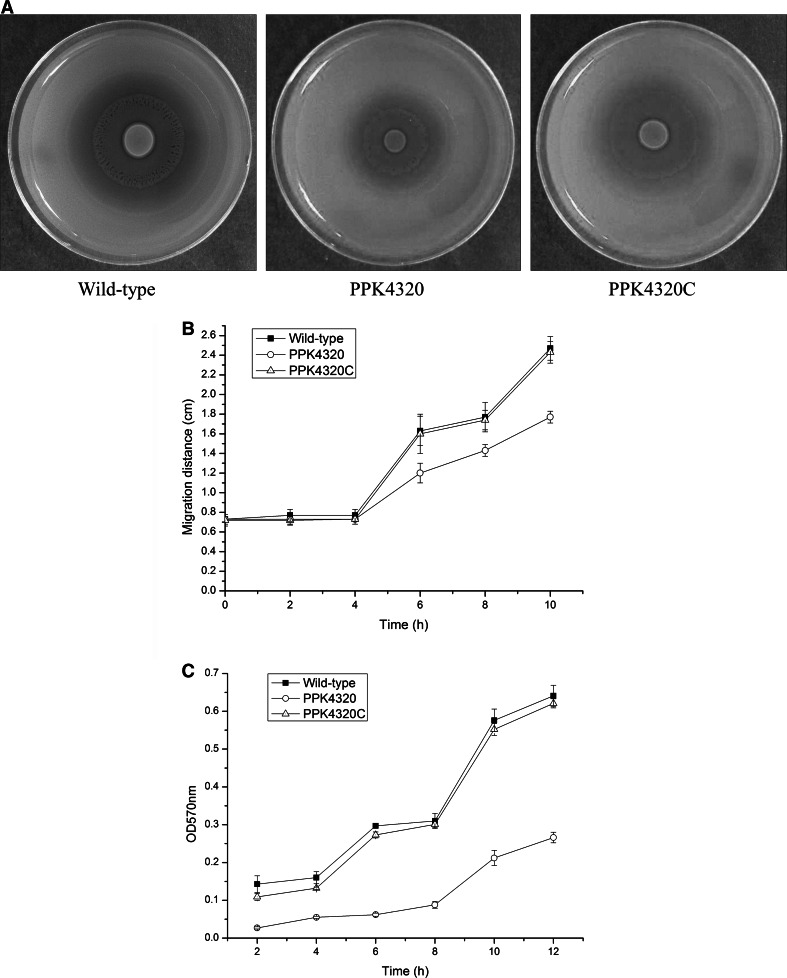
Examination of swarming motility and biofilm formation. **a** Halo images of the bacteria swarming on blood agar plates. Aliquots (5 µL) of overnight cultures were inoculated onto the centers of the plates and cultured at 37 °C for 10 h. **b** *Line* graph showing the swarming migration distances of the wild-type, PPK4320 and PPK4320C. Aliquots (5 µL) of overnight cultures were inoculated onto the centers of the swarming plates. The plates were incubated at 37 °C, and the migration distances were measured hourly after inoculation. The data represent the means of three independent experiments with standard deviations. **c** Biofilm formation for wild-type, PPK4320 and PPK4320C. The biofilm level of the wild-type, PPK4320 and PPK4320C was determined as described in “Materials and methods.” The optical density (570 nm) of the solution extracted with ethanol/acetone correlated with the level of biofilm formation

Swarming migration is a property of *P. mirabilis*. The motility capacity is an advantage for *P. mirabilis* colonization within the urinary tract, related to the expression of virulence factors and to the capacity to invade urothelial cells [21]. The migration distances of the wild-type and PPK4320C strain at different time points were significantly longer than those of the *ppk* mutant PPK4320 when cultured on the blood agar plates (*P* < 0.05) (Fig. 6a, b). This result indicates that *ppk* inactivation could impair the swarming motility of *P. mirabilis*. Because biofilm formation has been found to be related with survival in a host and indwelling device infections for *P. mirabilis* [22], biofilm formation was detected in this study. As shown in Fig. 6c, when *ppk* was interrupted, biofilm formation was significantly decreased, in contrast to those of the wild-type and *ppk* complementation strain (*P* < 0.01).

### *ppk* is involved in regulation of virulence factors expression in *P. mirabilis*

**Fig. 7:**
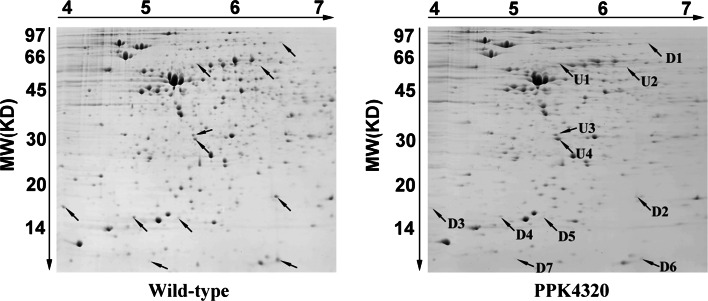
2-DE maps of the wild-type and the mutant PPK3420 bacteria. The up-regulated proteins are marked with U1–U4, and the down-regulated proteins are marked with D1–D7

To verify whether *ppk* is associated with protein expression regulation in *P. mirabilis*, we extracted the total protein from wild-type and *ppk* mutant bacteria for analysis using 2-DE. The experiment was performed three times independently. Only spots showing the same patterns in three independent runs were retained and quantified using ImageMaster V5.0 software. Seven down-regulated and four up-regulated spots were identified in the gels of the *ppk* mutant protein profiles compared with those of the wild-type bacteria (according to the criteria described in the Materials and Methods section) (Fig. 7). The selected spots were excised and identified by LC–MS/MS. The seven down-regulated proteins were TonB-dependent receptor, universal stress protein G, major mannose-resistant/Proteus-like fimbrial protein (MR/P fimbriae), heat shock protein, flagellar capping protein, putative membrane protein and multidrug efflux protein (Table 2). Additionally, the four up-regulated proteins were exported peptidase, repressor protein for FtsI, FKBP-type peptidyl-prolyl cis–trans isomerase and phosphotransferase. Among these proteins, MR/P fimbriae have been identified as critical adhesins for *P. mirabilis* colonization of the urinary tract [23]. Flagellar capping protein, also known as FliD, is associated with pathogen adhesion capacity and motility [24, 25]. Universal stress protein G and heat shock protein are both important for the protection of cells against stress [26, 27]. These alterations in protein expression may explain why the functions of *P. mirabilis* changed in their stress resistance, adhesion and migration capacities after *ppk* inactivation. We conclude that *ppk* is involved in stress tolerance and virulence of *P. mirabilis* by regulating the expression of the proteins mentioned above.

**Table 2:** List of protein spots identified by peptide mass fingerprinting

Spot ID	Access number	Mass (kDa)	Protein name
*Down*-*regulation*			
D1	gi|194682214	74.0	TonB-dependent receptor
D2	gi|197285310	15.4	Universal stress protein G
D3	gi|197284171	18.0	Major mannose-resistant/Proteus-like fimbrial protein
D4	gi|197284852	17.3	Heat shock protein
D5	gi|197285480	52.3	Flagellar capping protein
D6	gi|194684989	11.0	Putative membrane protein
D7	gi|197284044	42.2	Multidrug efflux protein
*Up*-*regulation*			
U1	gi|197284692	56.4	Exported peptidase
U2	gi|197286188	52.7	Repressor protein for FtsI
U3	gi|197286630	26.9	FKBP-type peptidyl-prolyl cis–trans isomerase
U4	gi|197284623	26.4	Phosphotransferase

## Discussion

Poly polyphosphate (Poly P) is a linear chain inorganic phosphate linked by “high-energy” phosphoanhydride bonds [28] and is present in all species in nature [28, 29]. As a major poly P synthetic enzyme, poly P kinase, which reversibly catalyzes the polymerization of the terminal phosphate of ATP into a poly P chain, is associated with stress resistance and virulence in many pathogens [30]. However, the role of PPK in the pathogenesis of UTIs caused by *P. mirabilis* remains unknown. To address this issue, we constructed the *ppk* isogenic insertional mutant of *P. mirabilis* HI4320 to test its biological functions using in vitro and in vivo models of UTI.

When the *ppk* gene was interrupted by using an isogenic insertion of group II intron method, the expression of the PPK protein was shown to be blocked. And the poly P production of *ppk* mutant PPK4320 was also reduced significantly compared to those of the wild-type and *ppk* complementation strain PPK4320C. The PPK4320 was considerably less adherent and invasive to the urothelial cells and significantly less virulent in the colonization of mouse bladders compared to the wild-type and PPK4320C. UTIs involving bacteria can provoke the body’s immune response and can induce the expression of inflammatory factors. IL-6 and TNF-α are both important cytokines released during acute UTI [18–20]. The results of the ELISA analysis showed that the in vivo expression levels of cytokine IL-6 and TNF-α were significantly reduced in the *ppk* mutant-infected bladders compared to the wild-type and *ppk* complementation strain-infected bladders. Histologic observation also demonstrated that the *ppk* mutant is ineffective in inducing bladder damage.

*Proteus mirabilis* encoding 17 putative fimbrial operons and five fimbrial types (ATFs, ambient-temperature fimbriae; MR/K, mannose-resistant Klebsiella-like; MR/P, mannose-resistant Proteus-like; NAFs, non-agglutinating fimbriae; and PMFs, *Proteus mirabilis* fimbriae) have been characterized [31]. Among the five types of fimbriae, MR/P contributes to adhesion and invasion of the uroepithelial cells and bladder and kidney colonization [32–34]. Flagellar capping protein, also called the hook-associated protein 2 (HAP2), facilitates the polymerization of endogenous flagellin at the tips of the growing flagellar filaments. Previous studies have reported that flagellar capping protein is involved in mucin adhesion and UTI [24]. Using a proteomics analysis, we demonstrated that *P. mirabilisppk* is involved in the production of MR/P. These results indicated that *ppk* inactivation leads to an attenuated virulence (including cell adhesion and invasion, bladder and kidney colonization) and induces a tissue inflammatory response, which may be due to modulation of the expression of MR/P and flagellar capping protein.

Swarming and biofilm formation are both important for *P. mirabilis* for causing catheter-mediated urinary tract infections. Swarming motility allows *P. mirabilis* to migrate across catheters, gaining entry to the urinary tract [1, 35]. The *ppk* mutant was deficient in swarming motility and had a decreased expression of flagellar capping protein. It has been previously reported that a mutation in the gene encoding flagellar capping protein prevented the assembly of normally synthesized FlaA flagellin monomers and resulted in the loss in motility and swarming differentiation [25]. We conclude that *ppk* is important for the swarming capacity of *P. mirabilis* by affecting the production of flagellar capping protein. The *ppk* gene has been identified to be related to the biofilm formation of the *P. aeruginosa* [8]. In this study, we also found that the biofilm formation of *P. mirabilis* was attenuated due to the *ppk* interruption.

During the UTI development process, it is important for the pathogens to endure the stressful environment of the urinary tract. We found that the *ppk* mutant is defective in surviving oxidative, osmotic and heat stress conditions compared to the wild-type bacteria. The proteomics analysis results demonstrated that universal stress protein G, heat shock protein and a putative membrane protein had low expression levels in the *ppk* mutant. Universal stress protein G belongs to the universal stress protein superfamily and is involved in oxidative stress resistance [26]. Heat shock proteins are a group of proteins induced by heat shock and are known to be involved in temperature stress adaption and the folding and unfolding of other proteins [27]. These proteins are likely involved in the insufficient stress adaptation conferred by the *ppk* mutation in *P. mirabilis*.

In summary, the major finding of this study is that the *ppk* gene, which is related to the poly P synthesis, plays an important role in stress tolerance and virulence of *P. mirabilis* in the urinary tract. However, the attenuation caused by the *ppk* mutation might not be merely the result of the loss of stress resistance, swarming migration, biofilm formation and urinary tract colonization capacities but rather might be caused by the inability to properly regulate the production of other proteins, including some important virulence factors that directly mediate the pathogenic process of *P. mirabilis*-induced UTI.
